# Boston Ivy-Inspired Disc-Like Adhesive Microparticles for Drug Delivery

**DOI:** 10.34133/2021/9895674

**Published:** 2021-05-17

**Authors:** Lijun Cai, Guopu Chen, Yuetong Wang, Cheng Zhao, Luoran Shang, Yuanjin Zhao

**Affiliations:** ^1^State Key Laboratory of Bioelectronics, School of Biological Science and Medical Engineering, Southeast University, Nanjing 210096, China; ^2^Zhongshan-Xuhui Hospital, The Shanghai Key Laboratory of Medical Epigenetics, The International Co-Laboratory of Medical Epigenetics and Metabolism, Ministry of Science and Technology, and Institutes of Biomedical Sciences, Fudan University, Shanghai 200032, China; ^3^Department of Rheumatology Immunology, Institute of Translational Medicine, The Affiliated Drum Tower Hospital of Nanjing University Medical School, Nanjing 210008, China

## Abstract

Microparticles with strong adherence are expected as efficient drug delivery vehicles. Herein, we presented an ingenious hydrogel microparticle recapitulating the adhesion mechanism of Boston ivy tendrils adhesive discs (AD) for durable drug delivery. The particles were achieved by replicating a silica colloidal crystal aggregates assembled in a droplet template after rapid solvent extraction. Due to their unique shape, the nanostructure, and the sticky hydrogel component, such novel microparticles exhibited prominent adhesive property to the wet tissue environment. It was demonstrated that the bioinspired microcarriers loading with dexamethasone had a good therapeutic effect for ulcerative colitis due to the strong adhesion ability for prolonging the maintenance of drug availability. These virtues make the biomimetic microparticles potentially ideal for many practical clinical applications, such as drug delivery, bioimaging, and biodiagnostics.

## 1. Introduction

Over the past years, tremendous progress has been made in particulate drug carriers for safer and more efficient drug delivery [1–7]. Among all, microparticles are recognized as one of the best candidates for controlled drug delivery because of their distinct advantages including the ease of production and characterization, high loading capacity, and low toxicity as compared with the nanosized systems [8–11]. For an optimal design, the geometrical features of the drug vehicles play an important role as it determines the adhesion and motion profile in the physiological environment [12, 13]. However, most microparticles, as fabricated via an emulsion template, take on a spherical shape. Due to the dinky contact area with a target biological substrate, the adhesion propensity and binding probability of these particles are usually insufficient, which hinders the long-term maintenance of drug concentration, and thus reducing the overall drug delivery efficiency [14]. Although efforts have been directed towards creating nonspherical vehicles including the rod or discoidal-like ones, their adhesion ability remains unsatisfactory, particularly in the dynamic fluid environment such as the esophagus and gastrointestinal tract. Therefore, emerging requirements have been raised to developing microparticles with a more elaborate, multiscale structural design, and strong adhesion performance as drug delivery carriers.

**Figure 1:**
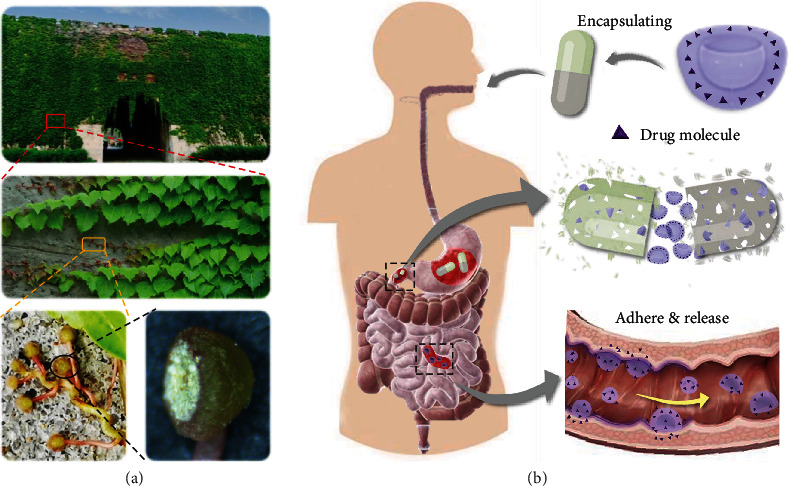
Scheme illumination of adhesive disc microparticles. (a) Images of the Boston ivy and their adhesive disc. (b) Scheme of the AD-like microparticles serving as adhesive drug carriers for prolonged drug release.

In this paper, inspired by the adhesive discs (AD) of Boston ivy tendrils, we present a novel microparticle with prominent adhesive ability as carriers for drug delivery, as schemed in Figure 1(a). Boston ivy is a wall-climbing plant capable of vertical growth to an extraordinary height by strongly affixing its adhesive discs in its tendrils to the substrate even under harsh weather conditions [15]. Such long-time wall-attachment ability attributes to not only the disc shape but also the presence of a microscale granular texture and the secretion of adhesive compounds, to a large extent [16, 17]. This feature provides a biomimetic strategy of designing an AD-like material for adhesion-related applications. However, approaches which are capable of recapitulating the multiscale structural and functional features of AD remain elusive due to the complexity of fabrication. In contrast, assembling of colloidal particles is a promising method because each particle serves as a separate microscale structural unit, while the final shape anisotropy of the assembly can be well-controlled by tuning the parameters [18–23]. Nevertheless, such a strategy has seldom been employed in the design of complex drug carriers.

Herein, we provided an AD-like microparticle as durable drug carriers by using functional hydrogel to replicate AD-like colloidal crystal templates, which were generated via a droplet-templated assembly process with rapid solvent extraction in the microfluidic system (Figure 1(b)). Due to the AD-mimetic multiscale structure feature, the resultant particles facilitated effective adhesion to the target objects. To further consolidating the adhesion ability, sticky molecules or charged molecules were added to the hydrogel matrix. Besides, magnetic nanoparticles or other ingredients could be incorporated into the particles to generate multifunctions such as magnet-assisted collection. Benefiting from their unique morphology, the nanostructure, and the sticky hydrogel component, the resultant AD-like hydrogel microparticles possessed strong adhesion ability, which was confirmed through an *in vitro* and *in vivo* affinity test to the colon epithelium surface. It was demonstrated that by loading dexamethasone into these adhesive particles, they could more firmly anchor to the area of the inflamed lesion to extend the drug release time compared to the spherical particles. This was due to the larger contact area and better interaction between the nanostructures and molecules, which contributed to an enhanced therapeutic effect for the treatment of ulcerative colitis *in vivo*. Therefore, the present bioinspired adhesive disc microparticles hold great potential in clinical applications, including in drug delivery and the associated therapeutics.

## 2. Results

**Figure 2:**
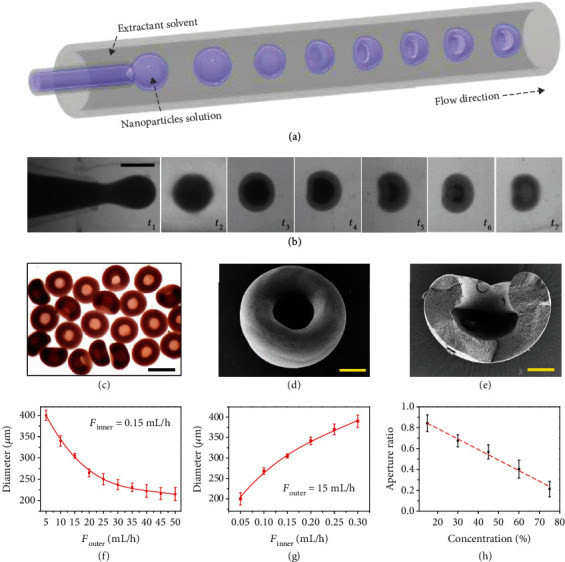
Fabrication and characterization of the AD-like microparticles. (a) Scheme of the generation process of the AD-like microparticles from coflow geometry microfluidics. (b) Images of the formation of the AD-like microparticles from coflow geometry microfluidics. The scale bar is 250 *μ*m. (c) Bright-field microscopic images of the resultant AD-like microparticles. The scale bar is 250 *μ*m. (b–e) SEM images of the resultant AD-like microparticles. The scale bar is 60 *μ*m in (d) and 55 *μ*m in (e). (f) Statistic analysis of the particle diameter at different velocity of the outer phase. (g) Statistic analysis of particle diameter at different velocity of the inner phase. (h) Statistic analysis of particle aperture ratio from different concentrations of nanoparticles.

In a typical experiment, the AD-like microparticles were obtained by confined assembly of colloidal crystals in a droplet template by rapid solvent extraction. The monodisperse droplets containing silica colloid nanoparticles were first generated from a microfluidic system, as schemed in Figure 2(a). The microfluidic device consisted of two capillaries intersecting at a junction to form a coflow geometry (Figure S1), where an aqueous suspension of silica nanoparticles was set as the inner phase and the solvent extractant was set as the outer phase. The aqueous solvent in the droplet was rapidly removed by the organic extractant and the silica colloids gradually self-assembled into an AD-like morphology due to the unbalanced extraction ratio [24, 25], as shown in Figures 2(b) and 2(c). To be specific, the deformation of the droplets was ascribed to that the concentration of the nanoparticles in the center of droplets was lower than that around the surface due to the rapid extraction.

Such morphology was further verified by the scanning electron microscope (SEM), resultant images showing the top and cross-sectional views (Figures 2(d) and 2(e)). It was worth mentioning that the diameters of the particles could be regulated by adjusting the velocity of the inner phase and outer phase, as depicted in Figures 2(f) and 2(g). Optimally, the flow rates of the inner phase and the outer phase were set as 0.15 mL/h and 15 mL/h in consideration of system stability and proper size of the particles. The amount of resultant particles per hour was counted and is shown in Figure S2. Furthermore, it was found that the morphology could be tuned by adjusting the concentration of silica colloid suspension. As shown in Figure 2(h), the aperture ratio of the formed particles decreased with the increase of silica's concentration in the original droplets. The definition of aperture ratio is schemed in Figure S3.

**Figure 3:**
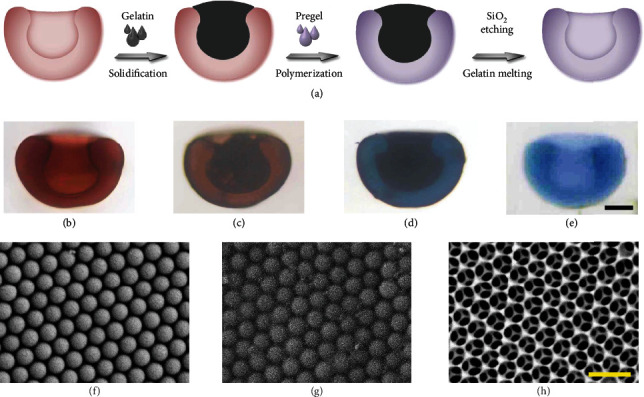
Fabrication and characterization of the AD-like hydrogel particle. (a) Scheme of the fabrication process of the AD-like hydrogel particle by replicating the structure of the AD-like microparticles. (b–e) Bright-field images of the replicating process and the intermediate product during each step. The scale bar is 70 *μ*m. SEM images of the nanostructures of the (f) silica colloidal crystal, (g) hydrogel infiltrated colloidal crystal, and (h) inverse opal hydrogel particle after the removal of the silica, respectively. The scale bar is 500 *μ*m.

To extend the bio-application of these AD-like particles, a biocompatible hydrogel, methacrylated gelatin (GelMa), was employed to replicate the unique morphology [26, 27]. The experimental process is depicted in Figures 3(a)–3(e), where black nanoparticles and blue dye were mixed into the melted gelatin solution and the pregel solution, respectively, to visualize the process. Firstly, the AD-like microparticles were immersed in a melted gelatin solution. The viscous gelatin solution could only fill the microscale cavity of the microparticles but not fill the nanovoids among the silica nanoparticles. The gelatin was solidified after cooling, and the extra gelatin on the surface was mechanically removed apart from that in the cavity. Then, the microparticles with gelatin in the cavity were dried and then soaked in a polymerizable pregel solution that could fill the nanovoids between the nanoparticles. Subsequently, ultraviolet (UV) light was utilized to polymerize the pregel, and the extra hydrogel on the surface was peeled off. Finally, the AD-like hydrogel microparticles were obtained by using hot hydrofluoric acid (HF) solution to etch the silica and melt the gelatin. The nanostructures of the intermediate products in each step were characterized by SEM and are shown in Figures 3(f)–3(h). It could be found that the silica nanoparticles self-assembled into a hexagonal close-packed arrangement after solvent extraction. As shown in Figure 3(g), the pregel successfully filled the voids between the nanoparticles. The ultimate AD-like hydrogel particles presented a periodically ordered 3D inverse opal structure as it was replicated from the colloidal crystal microparticles.

**Figure 4:**
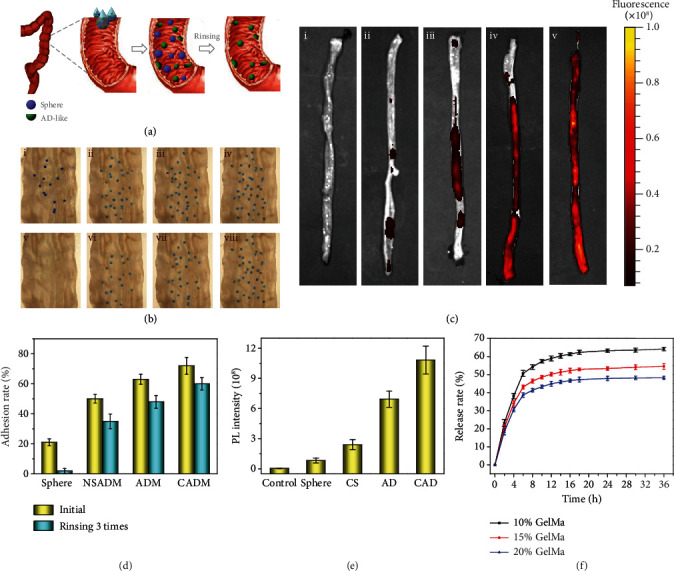
Test about the adhesive ability of the particles in vitro and in vivo. (a) Scheme of the *in vitro* test about the adhesive ability of the particles. (b) Images of the *in vitro* test about the adhesive ability of the particles after the first flow of the (i) spherical particles, (ii) AD-like particles without nanostructure, (iii) AD-like particles, and (iv) charged AD-like particles and their corresponding images after rinsing 3 times (v–viii). (c) Fluorescence images of the colon from mice with ulcerative colitis that received enema of (i) control, (ii) spherical particles, (iii) charged spherical particles, (iv) AD-like particles, and (v) charged AD-like particles. (d) Statistic analysis of *in vitro* test about the adhesive ability of the particles. NSADM: AD-like particles without nanostructure; ADM: AD-like microparticles; CAD: charged AD-like microparticles. (e) Statistic analysis of the fluorescence signal of *in vivo* test about the adhesive ability of the particles in correspondence with (c). CS: negatively-charged sphere; AD: AD-like microparticles; CAD: negatively charged AD-like microparticles. (f) The cumulative release profiles of Dex from the AD-like hydrogel microparticles.

We speculated that such nonspherical AD-like hydrogel microparticle could show better adhesive ability due to the joint effects of the larger contact area with the target surface and its nanostructure which enhances the local friction. To test this hypothesis, we first compared the adhesive ability of the AD-like hydrogel microparticles with that of the spherical hydrogel microparticles and the AD-like particles without nanostructures through an *in vitro* attachment test to the colon epithelium mucosa of healthy rats. A sample of a constant number of microparticles in the PBS buffer solution flowed through the colon epithelium surface. It was found that some particles remained on the surface due to adherence ability while the others slipped away with the flow. Then, additional PBS flow without microparticles was employed to flush through the surface for three times. The adhesion rate was calculated by counting the microparticles remained on the colon epithelium surface. It was worth mentioning that blue dye was added to visualize the particles. As illustrated in Figures 4(a) and 4(b), the spherical particles almost slipped away with the PBS flow, while the AD-like particles still anchor strongly to the surface even after flushing, indicating that the larger contact area benefited the adherence. In addition, the AD-like particles without the nanostructure showed a lower adhesion rate than those with the nanostructure on the surface. The adhesion rate of the AD-like hydrogel microparticles with the inverse opal structures was 45%, which was prominently higher than that of the other two groups, indicating that both the large contact area and the nanostructure contributed to the better adherence (Figure 4(d)). Then, we optimized the morphology of the particle by testing the adhesive ability of the hydrogel microparticles with different aperture ratios. As shown in Figure S4, the AD-like hydrogel particles with an aperture ratio of 45% showed the optimized adhesiveness. Moreover, considering that the colon epithelium surface of the healthy rats was negatively charged, we added positively charged molecules, to the hydrogel to further reinforce the adhesive ability. It was proved that, with the integration of the charged molecule additives, the microparticles exhibited even stronger adhesive performance with an adhesive rate of 61.7% (Figure 4(d)). To facilitate flexible control of the microparticles, magnetic nanoparticles were incorporated into the pregel solution in the fabrication process to impart the microparticles with a magneto-responsive feature, as shown in Figure S5. We thus foresee that those nonadhered microparticles could be separated under a magnetic field.

To test the adhesion ability of the AD-like hydrogel particles under a pathological environment, we took the ulcerative colitis (UC) tissue as an example. UC is a common inflammatory bowel disease which is mainly caused by lesions in the mucosa and submucosa of the large intestine [28, 29]. As the damaged epithelial surface is positively charged [30–32], we fabricated the AD-like particles with negatively charged molecules embedded in the hydrogel matrix, accordingly, to optimize the adhesive ability to the inflamed lesions. The zeta potential (-44.4 mV) confirmed that the hydrogel particles were imparted with a negative charge. The biocompatibility of the hydrogel was proved by culturing them with NIH-3T3 cells (Figure S6-S7). Then, the adhesive ability was verified *in vivo* by testing on the colon epithelium in the rats with dextran sulfate sodium (DSS) modeling. The rats with ulcerative colitis received a single enema with spherical microparticles without charge, spherical particles with negative charge, AD-like microparticles without charge, and AD-like microparticles with a negative charge. These rats were sacrificed 12 hours later, and the distal colon was removed for the analysis of adhesive ability, and the fluorescence retention resulting from the dye in the hydrogel was quantified. According to the fluorescence images in Figure 4(c), after 12 hours, there were almost no spherical particles left and only a few charged spherical particles remained. By contrast, the fluorescence signal of the AD-like particles group was much stronger than that of the spherical one, indicating that the AD-like particles exhibited better adherence. Furthermore, the fluorescence signal of the charged AD-like particles group was even stronger than that of the noncharged ones, and the particles stuck at some parts of the distal colon, which was ascribed to the electrostatic interaction between the negatively charged hydrogel and the positive charged damaged epithelial surface (Figure 4(e)). To evaluate the stability, we then collected the remnant AD-like particles from the distal colon and observed their structure. It was found that those particles swelled in the colon environment and their morphology showed little difference from the initial particles, indicating good stability in the colon environment (Figure S8). Together, these results provided evidence that the charged AD-like hydrogel was capable of preferential adherence in the complex pathological environment.

To test the AD-like hydrogel microparticles serving as drug vehicles, their performances in drug delivery were investigated through *in vitro* experiments. We loaded the hydrogel microparticles with dexamethasone (Dex), which is a kind of corticosteroid medicine proved to be utile for the treatment of UC induced by DSS [33–35]. It was worth mentioning that Dex could be well-loaded by physical adsorption to the hydrogel matrix of the AD-like microparticles due to the nanopores that provided a large surface-to-volume ratio. As shown in Figure S9, with the increase of the concentration of GelMa, the DL rate decreased gradually, which was attributed to the decrease of the porous ratio of the hydrogel. To investigate the release kinetics of Dex in the hydrogel microparticles, a group of the drug-loaded AD-like hydrogel particles with different solid content was emerged in a simulated human physiological environment of the intestinal fluid at 37°C. The cumulative release profiles of Dex from the AD-like hydrogel microparticles are shown in Figure 4(f). An initial burst release occurred within 10 hours, followed by a continuous profile that eventually reached the maximum. It was worth noting that the release rate of Dex from the particles increased with the decrease of the solid content on the GelMa hydrogel. Although the lower solid content of the hydrogel particle resulted in a weaker mechanical strength, a value of 10% is enough to maintain the integrity of the inner nanostructure. Besides, the particles with a higher solid content contributed to improved adsorption. Therefore, a range of 10% ~20% of the solid content was set reasonable for the AD-like hydrogel particles to serve as an ideal candidate as a drug delivery carrier.

**Figure 5:**
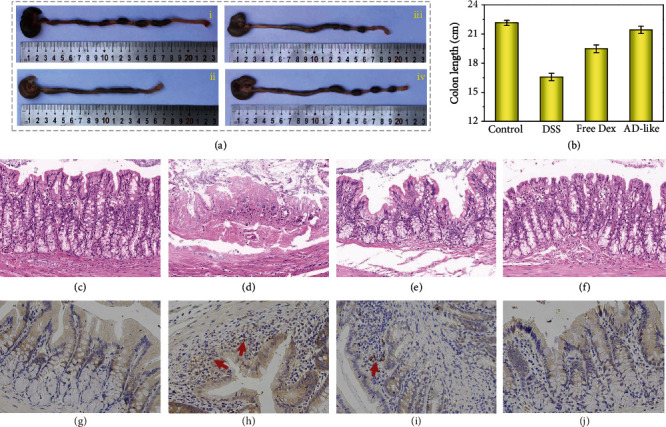
Analysis of the therapeutic effect. (a) Images of colons from healthy mice (i) and mice with DSS modeling that received enema with PBS (ii), free Dex (iii), and AD-like particles loading with Dex (iv). (b) Statistic analysis of the colon length from different groups. Representative H&E histology images of healthy mice (c) and mice with DSS modeling that received enema with PBS (d), free Dex (e), and AD-like particles loading with Dex (f). Representative images of MPO activity in colonic sections of healthy mice (g) and mice with DSS modeling that received enema with PBS (h), free Dex (i), and AD-like particles loading with Dex (j).

**Figure 6:**
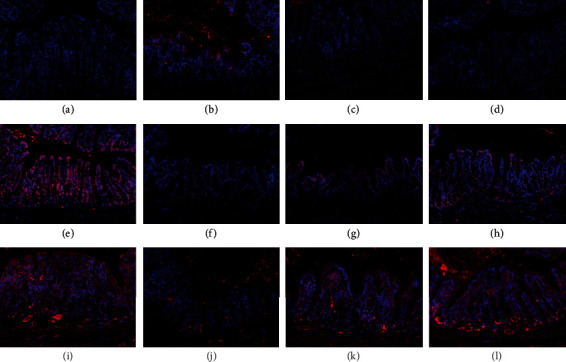
Analysis of the therapeutic effect. Representative images of expression of IL-6 in colonic sections of healthy mice (a) and mice with DSS modeling that received enema with PBS (b), free Dex (c), and AD-like particles loading with Dex (d). Representative images of Occludin immunostaining in colonic sections of healthy mice (e) and mice with DSS modeling that received enema with PBS (f), free Dex (g), and AD-like particles loading with Dex (h). Representative images of Zo-1 immunostaining and in colonic sections of healthy mice (i) and mice with DSS modeling that received enema with PBS (j), free Dex (k), and AD-like particles loading with Dex (l).

Integrating their excellent properties in adherence and drug delivery, the AD-like hydrogel microparticles were expected to shed new light on biomedicine. To verify the practical therapeutic effect of the drug-loaded AD-like particles, the rats with DSS modeling received an enema with PBS, free Dex solution, and the Dex-loaded AD-like hydrogel microparticles, respectively, in day 1 and day 3. On day 5, all mice were sacrificed for evaluation of therapeutic efficacy. Since DSS would lead to congestive and edematous colonic membrane, thus resulting in colon shortening [36, 37], colon weight gaining, and body weight loss, we recorded the colon length, colon weight, and body weight of the mice that received different enema treatments. There was an evident statistic distinction between the lengths of colons (Figures 5(a) and 5(b)). To be specific, the colon length of the mice with DSS modeling who received enema of Dex-loaded AD-like microparticles was longer than that receiving PBS and free Dex, which showed little difference with that of the healthy mice. In addition, the colon/body weight ratio is presented in Figure S10. The colon/body weight ratio of the mice with DSS modeling who received enema Dex-loaded AD-like microparticles decreased most, presenting a closer value to the healthy mice compared with the other two groups. The characteristics of DSS are infiltration of the colon lamina propria with neutrophils and mononuclear inflammatory cells, crypt hypertrophy, and superficial erosions. In addition, we demonstrated from the hematoxylin and eosin (H&E) histology images (Figures 5(c)–5(f)) and histopathology scores (Figure S11) that histological inflammation was significantly diminished in mice that received enema with the Dex-loaded AD-like hydrogel microparticles compared to those that received enema with the equivalent amount of free Dex solution. Furthermore, myeloperoxidase (MPO) activity (Figures 5(g)–5(j)) and expression of interleukin-6 (IL-6), IL-1, and factor-*α* (TNF-*α*) (Figures 6(a)–6(d) and Figure S12) in the colon were detected, all of which are important indicators for colitis treatment. We found a decrease of the both indicators in mice that received treatment of AD-like particles compared with the other two experimental groups. Furthermore, immunofluorescence staining also demonstrated that the DSS modeling led to the reduced Occludin and Zo-1 protein expression at the epithelial tight junctions (Figures 6(e)–6(l)). The presence of the Dex-loaded AD-like hydrogel particles recovered the Occludin and Zo-1 protein expression to a greater extent than the free Dex. All the above-mentioned results demonstrated that the Dex-loaded AD-like microparticles showed an efficient therapeutic effect on the treatment of UC with their prominent adhesive property and efficient drug delivery performance, indicating potential in practical clinical application.

## 3. Discussion

In summary, inspired by the AD of Boston ivy, we have proposed an ingenious kind of AD-like hydrogel microparticles with strong adherence to the tissue for durable drug delivery. The AD-like hydrogel microparticles were obtained by replicating a silica colloidal crystal cluster generated from a droplet template after rapid solvent extraction. The AD-like shape, the interconnected surface patterning nanostructure, and the surface charge contributed altogether to the better adhesion performance of the resultant particles compared with traditional spherical microparticles, which was demonstrated both *in vitro* and *in vivo.* Benefiting from the adhesiveness feature, the particles served as drug vehicles for the durable drug delivery of Dex for the treatment of UC. It was demonstrated that the Dex-loaded AD-like hydrogel microparticles could effectually prolong the local drug availability and indicated efficient therapeutic results with minimum of systemic side effects. Therefore, the AD-like hydrogel microparticles exhibited unprecedented merits and are predicted to play a potential role in sustained drug delivery, bioimaging, and diagnostics.

## 4. Materials and Methods

### 4.1. Materials

The gelatin and 2-hydroxy-2-methylpropiophenone (HMPP) were purchased from Sigma-Aldrich, Shanghai, China. The glyceryl triacetate and hydrofluoric acid (HF) and chitosan were obtained from Sinopharm Chemical Reagent Co., Ltd. Dexamethasone (Dex) and cellulose sodium were brought from Aladdin, Shanghai, China. The magnetic nanoparticles were purchased from Nanjing Nanoeast Biotech Co. Ltd. Dextran sulfate sodium (DSS) was obtained from Dalian Meilun Biological Technology Co., Ltd. The feeding needle was purchased from Braintree Scientific. The silica nanoparticles, methacrylated gelatin (GelMa), simulated intestinal fluid, and phosphate-buffered saline (PBS, 0.05 M, pH 7.4) were self-prepared. All buffers were self-prepared using water purified in a Milli-Q system (Millipore, Bedford, USA).

### 4.2. Preparation of Silica Nanoparticles

According to the Stöber method, tetraethyl orthosilicate (TEOS) was mixed dropwise to the solution of ammonium hydroxide (10 mL) and ethanol (300 mL) at 30°C with stirring (300 rpm). Because of the condensation and hydrolysis of TEOS, the nanoparticles grew gradually. To obtained particles with desired size, the mixture solution was sampled every half hour to measure their diameter through SEM.

### 4.3. Synthesis of GelMa

Firstly, 10 g Gelatin Porcine was melted in PBS solution to form 100 mL mixture at 60°C. Then, 8 mL methacrylic anhydride was added slowly into the solution dropwise under rotating at 60°C for 3 hours. Next, 392 mL preheated PBS was added into the resultant solution, followed by rotating at room temperature for 15 min. After that, the mixture solution was transferred into a dialysis membrane, and then, the dialysis membrane was placed in a container with 5 L pure water to carry out the dialysis at 40 to 50°C for 1 week. The water was changed 2 to 3 times a day. After the dialysis, the GelMa solution was filtered by using a sterile filter. Finally, GelMa was obtained after freeze drying.

### 4.4. Fabrication of AD-Like Colloidal Microparticle

To fabricate the AD-like colloidal microparticles, droplets containing different concentrations of silica nanoparticles were subjected to solvent extraction in a microfluidic channel where the silicon nanoparticles aqueous suspension was set as the inner phase and the solvent extractant was set as the outer phase. To be specific, the rates of inner phase and outer phase were set as 0.15 mL/h and 15 mL/h, respectively. During the rapid extraction, the sphere droplets turn to form the AD-like morphology. After all the solvent in droplets was removed, the AD-like colloidal microparticles were obtained. Finally, they were calcined at 800°C for 12 h in a muffle furnace to stabilize the structure.

### 4.5. Preparation of AD-Like Hydrogel Particles

The AD-like colloidal microparticles were firstly dried and exposed to 10 wt% gelatin solution at 45°C for 30 min. After the gelatin filled the cavity, the particles were cooled down to solidify the gelatin (10°C). The extra gelatin was mechanically removed by rubbing them softly by hand. The resultant particles were dried and then immersed in a mixed solution containing GelMa with different concentrations together with 1 vol% HMPP, for 30 minutes. Magnetic nanoparticles with an average diameter of 10 nm and a concentration of 4 mg/mL were added to the mixture for the construction of magneto-responsive particles. After the mixed pregel solution filled the nanopores, ultraviolet light was utilized to polymerize the pregel. Subsequently, redundant hydrogel on the surface of the templates was mechanically removed. The AD-like hydrogel microparticles were finally obtained by using hot HF at 45°C to etch the silica nanoparticles in the templates and melt the solid gelatin in the cavity. To fabricate charged particles, the charged molecules, including sodium alginate and chitosan aqueous solution, were simply mixed into the mixture of pregel.

### 4.6. Verification of Biocompatibility

NIH-3T3 cells were cultured with DMEM composed of 10% FBS and 1% penicillin-streptomycin in a humidified incubator with constant temperature (37°C) and 5% CO_2_. The hydrogel containing 20% GelMa and hydrogel containing 20% GelMa and 10% sodium carboxymethylcellulose were treated with sterilization by utilizing UV light irradiation overnight and successively washing with PBS solution several times before cell culture. All groups of hydrogels were cut into the same size, similar to the area of a single well of a 24-well plate. Then, the NIH-3T3 cells were cultured on the surface of the two kinds of hydrogels. NIH-3T3 cultured on the surface of glasses cut into the same size was set as the control group. The activity of NIH-3T3 cells cultured in two layers was investigated. The solution of MTT (5 *μ*g/mL) was prepared and filtered. After cells were cultured on the surface for every 12 h, the films were rinsed with PBS. 70 *μ*L MTT solution was added in 600 *μ*L DMEM medium and cultured for another 4 h at 37°C. Subsequently, 600 *μ*L DMSO was added to dissolve the formazan crystal in cells for further measuring the OD value after the removal of the solution in the 24-well plate. Four parallel experiments were performed for each sample. The morphology of cells was also imaged. After being cultured for 48 h, calcein AM (2 *μ*g/mL, 2 mL per well) was added into each well to stain the cells for 20 min at 37°C. After using PBS to rinse for three times, the cells were observed using an inverted fluorescence microscope.

### 4.7. In Vitro Microparticle Adhesion Experiments

Droplets of PBS buffer containing 50 spherical microparticles, AD-like charged hydrogel microparticles, AD-like hydrogel microparticles without charge, and AD-like charged hydrogel microparticles without nanostructure, respectively, were rinsed through the colon epithelium surfaces of healthy rats *in vitro*. After that, 1 mL of PBS without particles was employed to rinse the surface for additional three times. Then, the number of microparticles that remained on the colon epithelium surfaces was counted to calculate the retention rate.

### 4.8. The Establishment of Colitis of Mice

The colitis of mice in the experiment was induced by using dextran sulfate sodium (DSS) modeling. Mice were Sprague Dawley (SD) rats with a weight of 200 g ± 10 g. 4% DSS solution was utilized to feed the SD mice for 2 weeks, while the control SD mice were fed with normal water.

### 4.9. In Vivo Microparticle Adhesion Experiments

The adhesion tests were conducted on the colon epithelium in the SD rats with colitis, who received an enema with spherical hydrogel particles, charged spherical hydrogel particles, AD-like hydrogel particles, and charged AD-like hydrogel particles, respectively. Mice were fasted overnight, with each mouse receiving 500 *μ*L enema with the same amount of hydrogel microparticles in the following morning. After anesthetization to individual mouse, a flexible disposable feeding needle was advanced into the rectum 3 cm past the anus, with microparticles administered. Then, the catheter was removed, and the anus was kept closed manually for 1 min. The rats were sacrificed after 12 hours. The colon was taken out and then imaged freshly without any washing. Notably, Rhodamine B was added to the pregel for fluorescence imaging. The fluorescence signal intensity was quantified using Living Image software (version 4.3.1, PerkinElmer) in a standard-size ROI drawn around individual colon pieces. Background fluorescence intensity was determined as the average of three ROIs not containing any colon tissue and was subtracted from all specimens.

### 4.10. Drug Loading and Drug Release In Vitro

A standard curve of Dex solution was determined by a UV spectrophotometer at 240 nm (characteristics wavelength of Dex). The AD-like hydrogel particles were dried and immersed in a DEX solution with a concentration of 1 mg/mL overnight. To assay the drug loading (DL) rate of the hydrogel microparticles, 2 mg dried hydrogel microparticles were immersed in 1 mL Dex solution with concentration of 1 mg/mL for 24 h. The remaining Dex solution was collected to a test tube and volumed to 1 mL to determine the OD value for calculating the weight of loaded Dex. The DL rate was calculated according to the following equation:

(1) $$\begin{array}{c} \text{DL}\%=\frac{\text{mass of drug loaded}}{\text{mass of hydrogel}}\times 100\%. \end{array}$$

To detect the drug release kinetics of these hydrogels, the hydrogel particles were taken out the next morning after immersing. The Dex-loaded microparticles were settled in 1 mL of simulated intestinal fluid in centrifuge tubes and were incubated at 37°C and oscillated at 500 rpm for 30 hours. At every predetermined time interval, 350 *μ*L of the supernatants was taken out to another centrifuge tube and assayed by an ultraviolet spectrophotometer. An equivalent volume (350 *μ*L) of fresh simulated intestinal fluid was added to each tube at each time point after assaying.

### 4.11. The Effects of Drug-Loaded AD-Like Microparticles on Colitis

The mice with colitis were randomized to four experimental groups: (i) healthy mice, (ii) mice with DSS receiving no enema, (iii) mice with DSS receiving free Dex enema, and (iv) mice with DSS receiving Dex-loaded AD-like hydrogel enema. Enemas were administered on day 1 and day 3 after fasting the mice overnight as described. All mice were sacrificed for histopathological analysis on day 5. Colons were isolated, fixed in 4% paraformaldehyde, and embedded in paraffin. Standard H&E-stained sections, immunohistochemistry of MPO, and immunofluorescence of IL-6, IL-1, and TNF-*α*, Occludin immunostaining, and Zo-1 immunostaining were examined. Colon length and colon weight of the mice were measured as additional parameters of disease activity.

### 4.12. Characterization

Optical images of the AD-like particles were captured by a stereomicroscope (JSZ6S, Jiangnan novel optics) equipped with a CCD camera (Oplenic digital camera). The cross-sectional microstructure of the microparticles was characterized by a field emission scanning electron microscope (FESEM, Ultra Plus, Zeiss). The fluorescence images of particles were taken by IVIS Spectrum (PerkinElmer). The measurement of drug release kinetics was taken out by an ultraviolet spectrophotometer (Agilent Cary 60).

## Data Availability

The data used to support the findings of this study are available from the corresponding author upon request.

## Supplementary Materials

Supplementary Materials Figure S1: illustration of the microfluidic device for fabricating AD-like microparticles. Figure S2: statistics of the number of resultant particles generated per hour. The error bar represents the standard deviation from ten independent experiments. Figure S3: scheme of the measurement of the aperture ratio of the AD-like particles. Figure S4: statistic analysis of the adhesive ability of particles with different aperture ratio. Figure S5: images showing that the magnetic nanoparticle-incorporated AD-like particles were collected into one side of a vial by the magnet. Figure S6: fluorescence images of NIH-3T3 cells cultured on the glass (a), GelMA (b), and the mixture hydrogel of GelMa and sodium alginate (c) for 36 h. Figure S7: MTT assay for the cells cultured on glass, GelMa, and the mixture hydrogel of GelMa and cellulose sodium (GC gel). Figure S8: microscopic images of the AD-like hydrogel microparticles before and after the enema. Scale bars are 250 *μ*m. Figure S9: statistic analysis of drug loading rate of the microparticles with different concentration of GelMa. The error bars represent the standard deviation from five independent experiments. Figure S10: statistic analysis of the colon/body weight from different groups. Figure S11: statistic analysis of histological scores for the H&E images from different groups. Figure S12: (a) representative images of expression of IL-1 in colonic sections of healthy mice (i) and mice with DSS modeling received enema with PBS (ii), free Dex (iii), and AD-like particles loading with Dex (iv). (b) Representative images of expression of TNF-*α* in colonic sections of healthy mice (i) and mice with DSS modeling received enema with PBS (ii), free Dex (iii), and AD-like particles loading with Dex (iv).
