# Supplementary material for: Boston Ivy-Inspired Disc-Like Adhesive Microparticles for Drug Delivery
Source: Research (Wash D C). 2021 May 17;2021:9895674. doi: 10.34133/2021/9895674 (PMC8153044; doi:10.34133/2021/9895674)
Supplement: Supplementary Materials — Figure S1: illustration of the microfluidic device for fabricating AD-like microparticles. Figure S2: statistics of the number of resultant particles generated per hour. The error bar represents the standard deviation from ten independent experiments. Figure S3: scheme of the measurement of the aperture ratio of the AD-like particles. Figure S4: statistic analysis of the adhesive ability of particles with different aperture ratio. Figure S5: images showing that the magnetic nanoparticle-incorporated AD-like particles were collected into one side of a vial by the magnet. Figure S6: fluorescence images of NIH-3T3 cells cultured on the glass (a), GelMA (b), and the mixture hydrogel of GelMa and sodium alginate (c) for 36 h. Figure S7: MTT assay for the cells cultured on glass, GelMa, and the mixture hydrogel of GelMa and cellulose sodium (GC gel). Figure S8: microscopic images of the AD-like hydrogel microparticles before and after the enema. Scale bars are 250 μm. Figure S9: statistic analysis of drug loading rate of the microparticles with different concentration of GelMa. The error bars represent the standard deviation from five independent experiments. Figure S10: statistic analysis of the colon/body weight from different groups. Figure S11: statistic analysis of histological scores for the H&E images from different groups. Figure S12: (a) representative images of expression of IL-1 in colonic sections of healthy mice (i) and mice with DSS modeling received enema with PBS (ii), free Dex (iii), and AD-like particles loading with Dex (iv). (b) Representative images of expression of TNF-α in colonic sections of healthy mice (i) and mice with DSS modeling received enema with PBS (ii), free Dex (iii), and AD-like particles loading with Dex (iv). [file 9895674.f1.docx]

Supplementary Materials


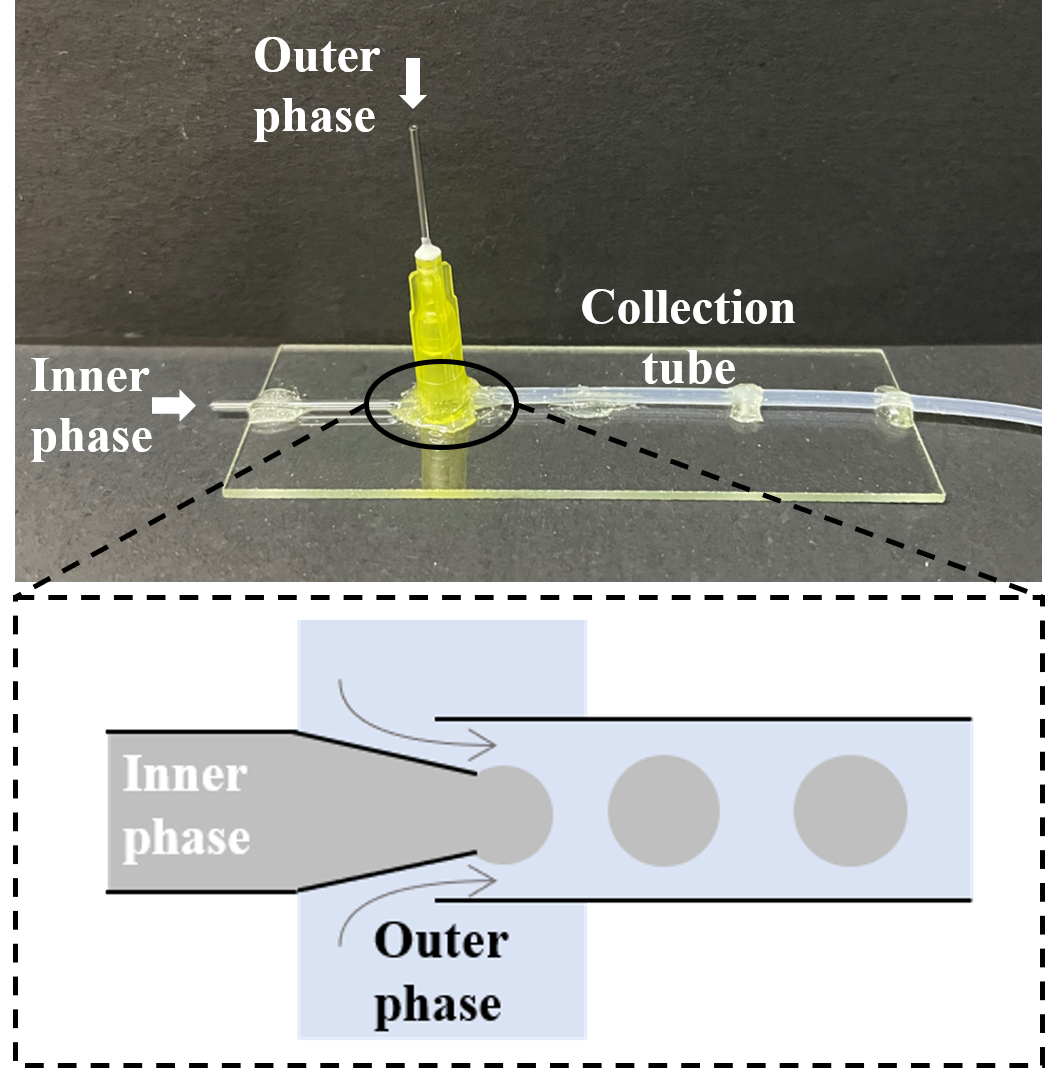


**Figure S1** Illustration of the microfluidic device for fabricating AD-like microparticles.

**
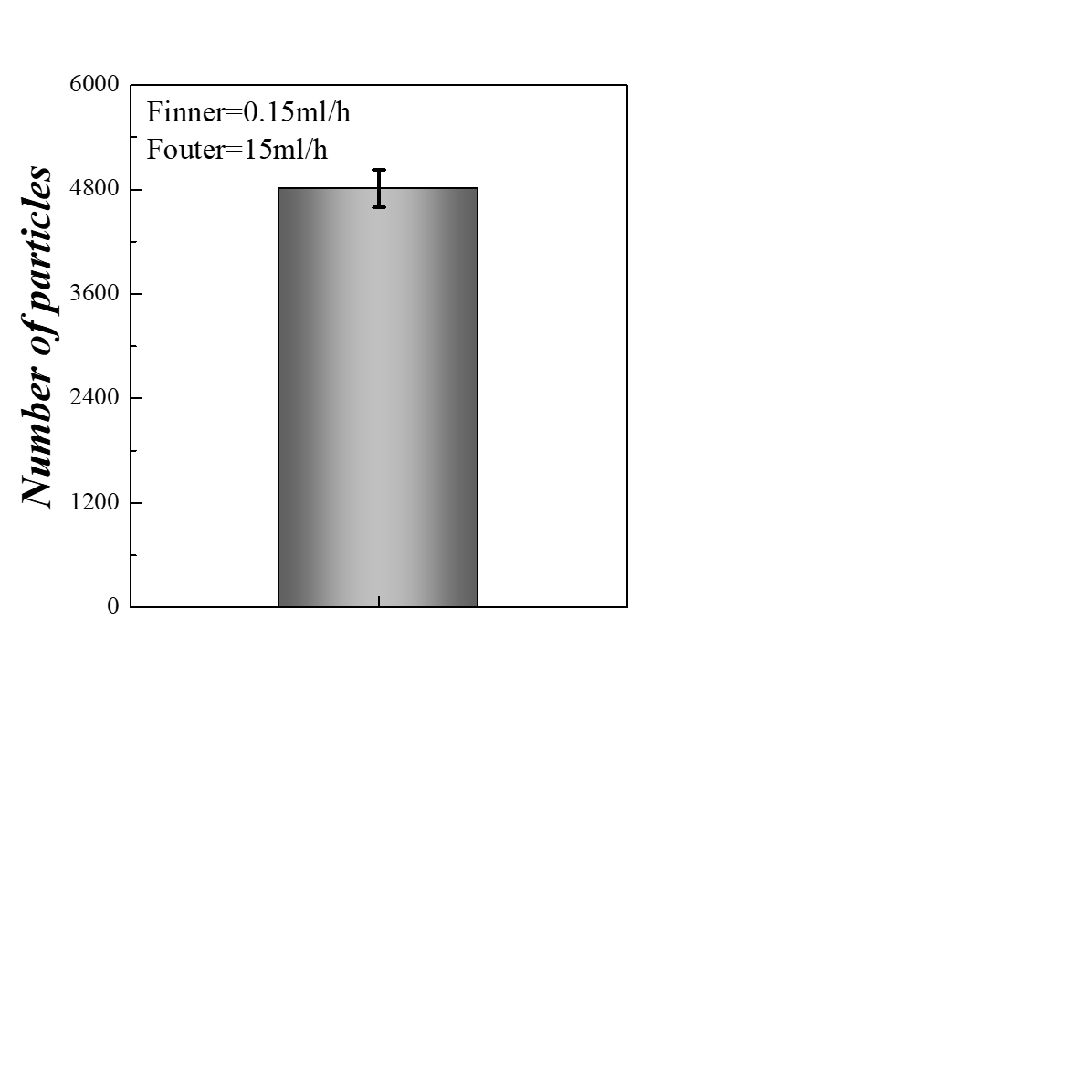
**

**Figure S2** Statistics of the number of resultant particles generated per hour. The error bar represents the standard deviation from ten independent experiments.


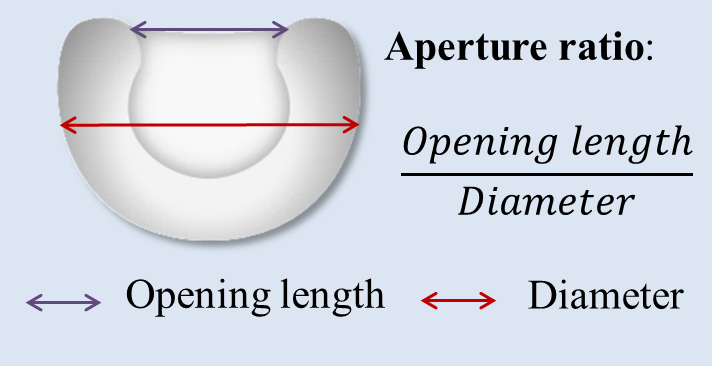


**Figure S3** Scheme of the measurement of the aperture ratio of the AD-like particles.

**
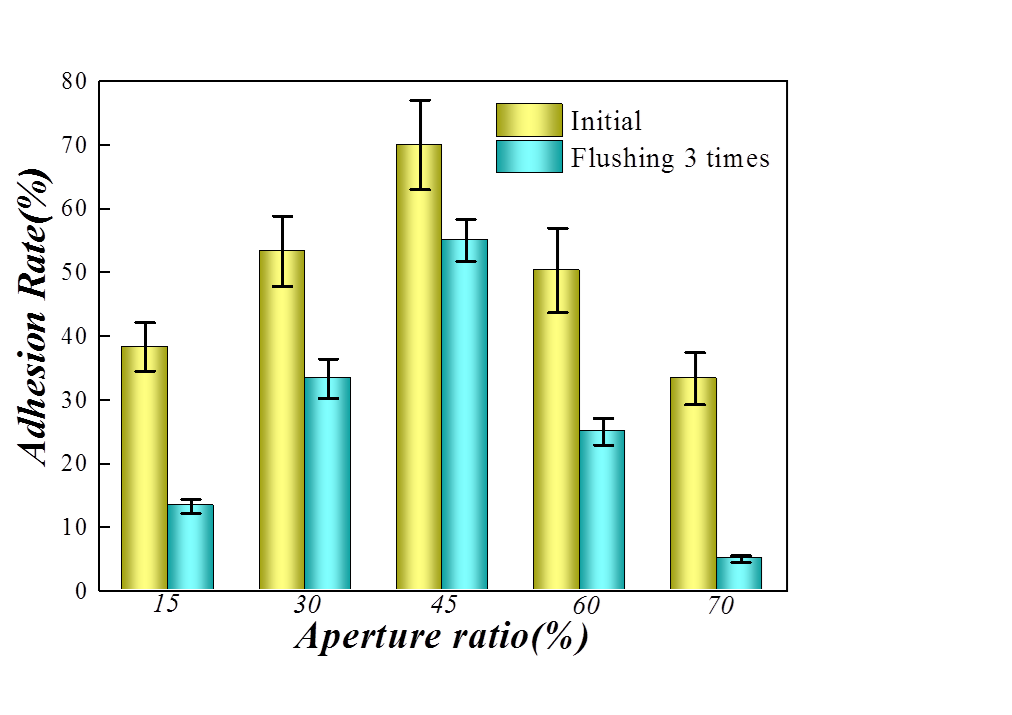
**

**Figure S****4** Statistic analysis of the adhesive ability of particles with different aperture ratio.

**
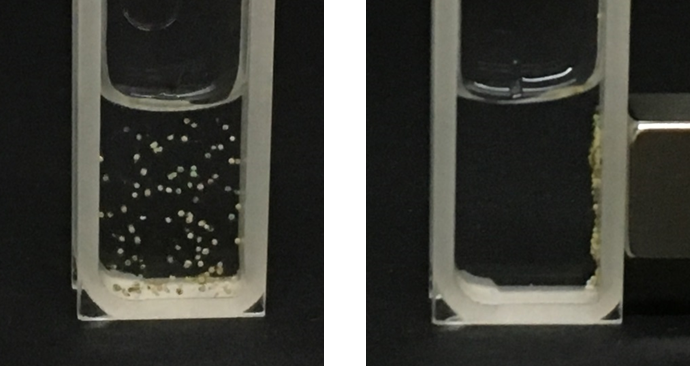
**

**Figure S5** Images showing that the magnetic-nanoparticles-incorporated AD-like particles were collected into one side of a vial by the magnet.

**
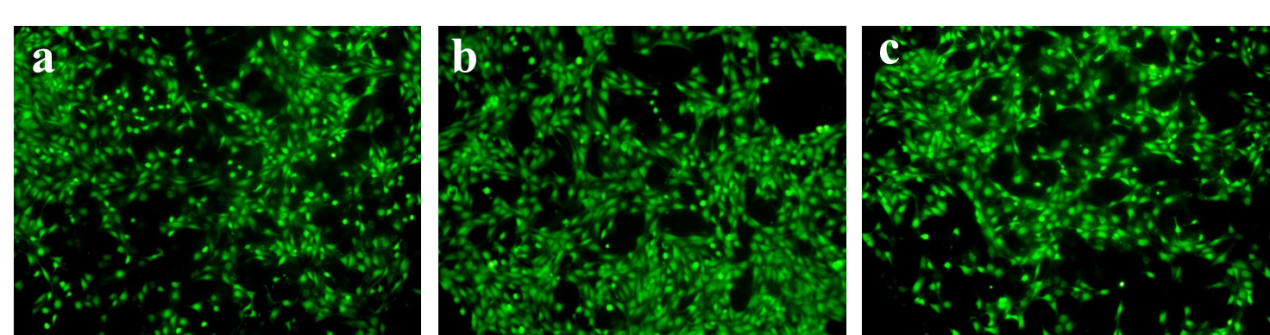
**

**Figure S6** (a-c) Fluorescence images of NIH-3T3 cells cultured on the glass(a), GelMA(b), and the mixture hydrogel of GelMa and sodium alginate(c) for 36 h.

**
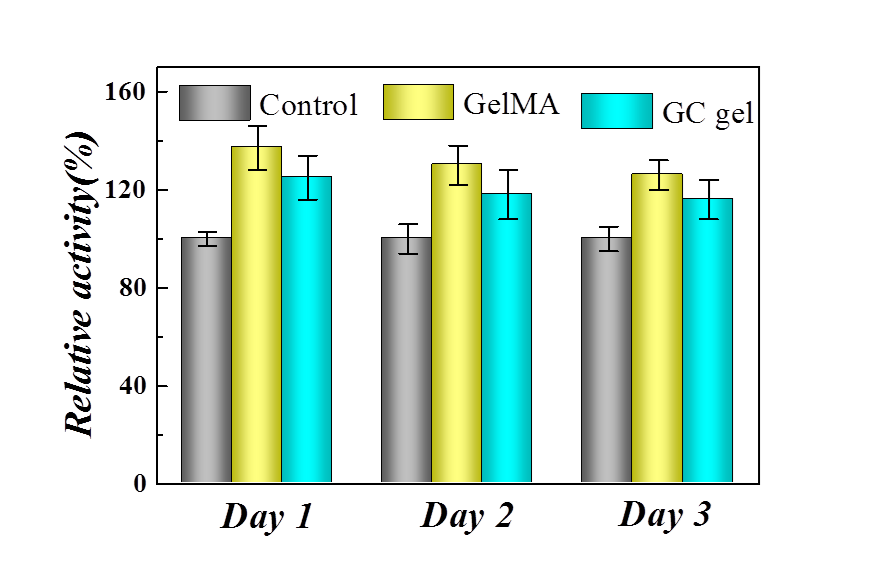
**

**Figure S7** MTT assay for the cells cultured on glass, GelMa and the mixture hydrogel of GelMa and cellulose sodium (GC gel).


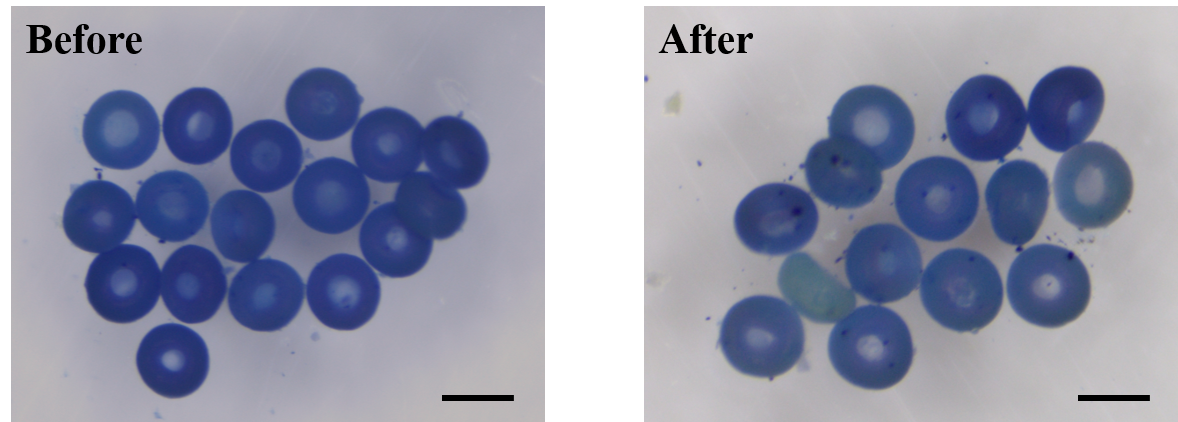


**Figure S8** Microscopic images of the AD-like hydrogel microparticles before and after the enema. Scale bars are 250μm.


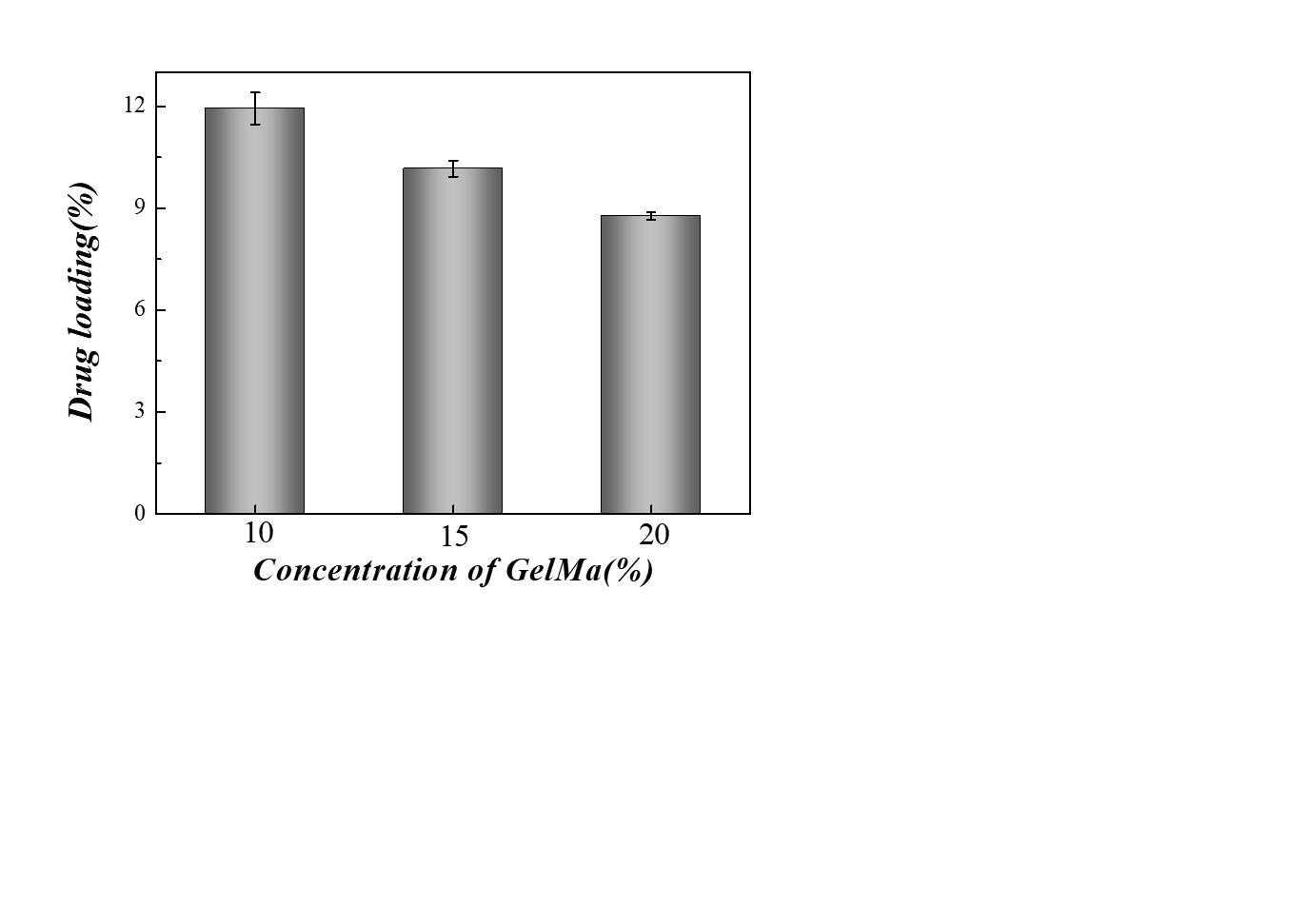


**Figure S9** Statistic analysis of drug loading rate of the microparticles with different concentration of GelMa. The error bars represent the standard deviation from five independent experiments.

**
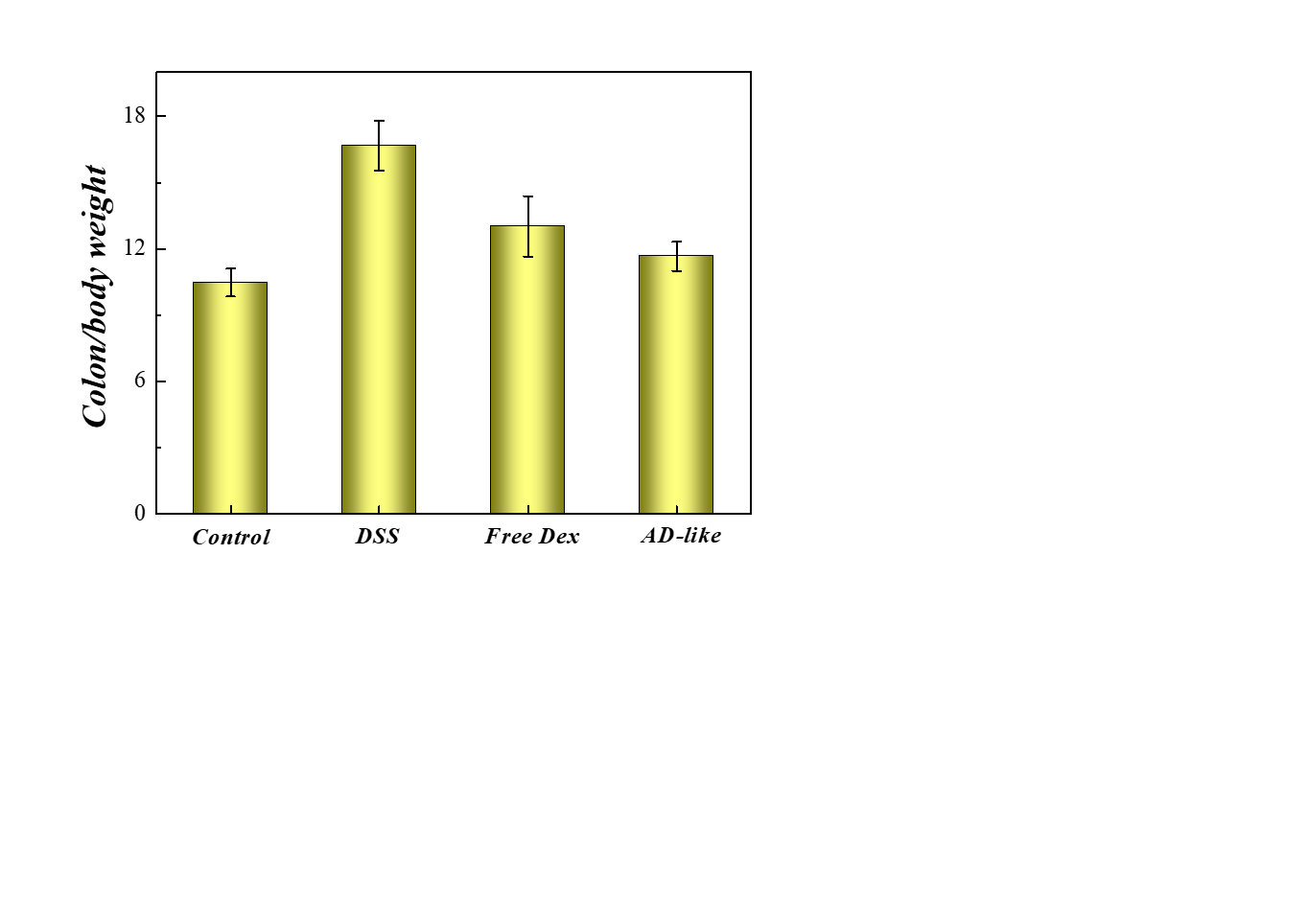
**

**Figure S10** Statistic analysis of the colon/body weight from different groups.

**
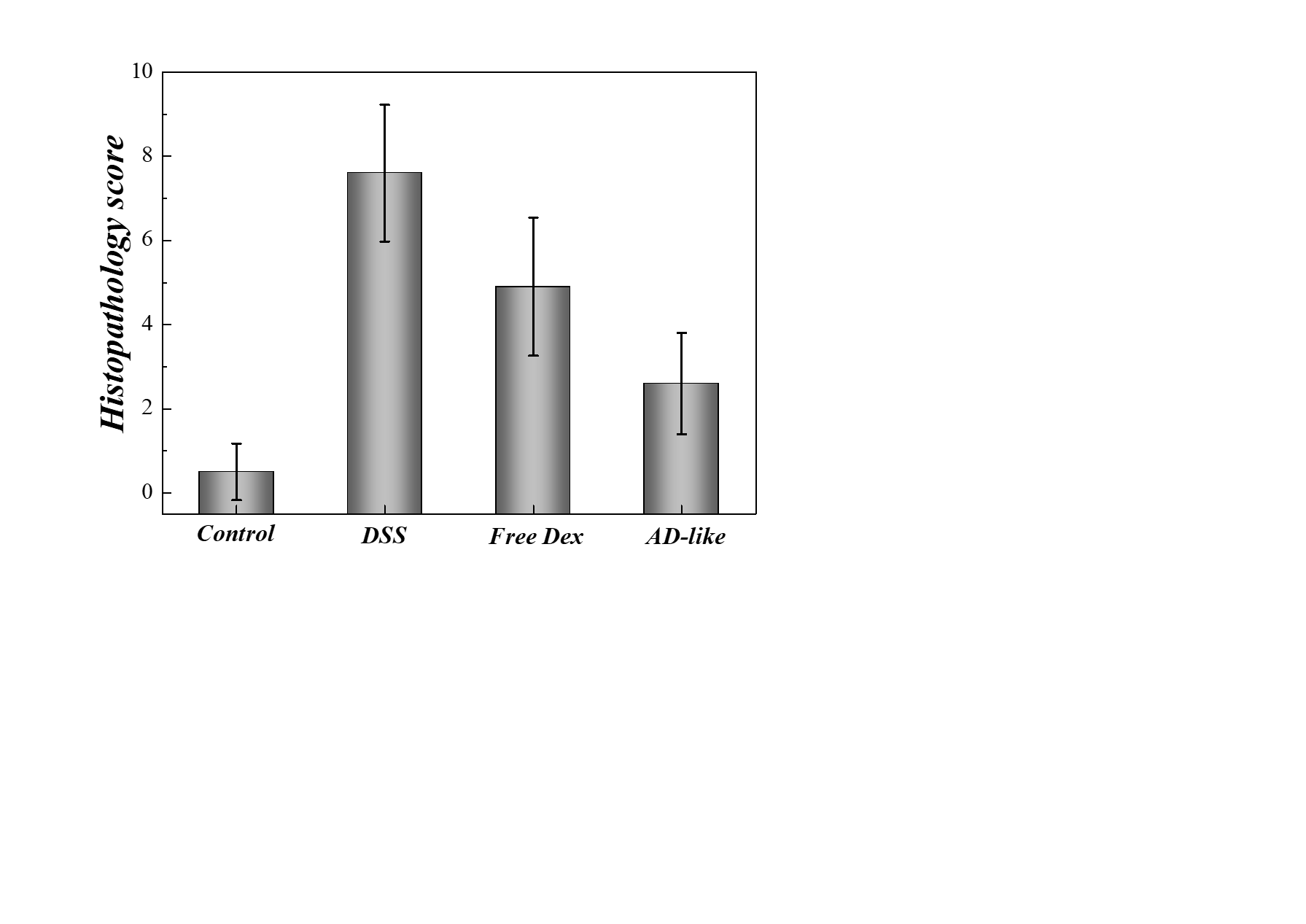
**

**Figure S11** Statistic analysis of histological scores for the H&E images from different groups.


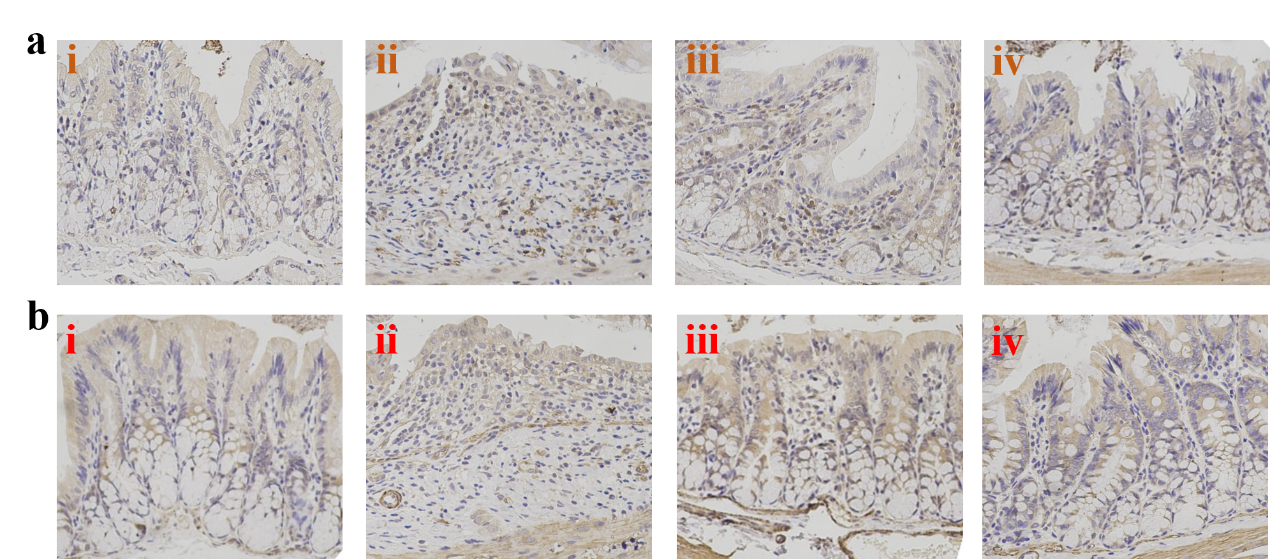


**Figure S12 (a)**Representative images of expression of IL-1 in colonic sections of healthy mice(i), mice with DSS modeling received enema with PBS(ii), free Dex(iii), and AD-like particles loading with Dex(iv). **(b)**Representative images of expression of TNF-α in colonic sections of healthy mice(i), mice with DSS modeling received enema with PBS(ii), free Dex(iii), and AD-like particles loading with Dex(iv).
